# Self-care and emotional engagement in therapeutic contexts: a predictive study of healthcare practitioners

**DOI:** 10.1186/s12875-026-03308-3

**Published:** 2026-04-09

**Authors:** Yahya Khatatbeh, Sawsan Al-Momen

**Affiliations:** Imam Mohammad Ibn Saud Islamic University (IMSIU), Riyadh, Saudi Arabia

**Keywords:** Mindful self-care, Clinical empathy, Healthcare practitioners, Predictive modelling, Structural equation modelling

## Abstract

**Background:**

Clinical empathy is essential to therapy quality, patient satisfaction, and practitioner well-being. Mindful self-care has been shown to prevent burnout, but less is known about its association with adaptive professional abilities such as clinical empathy. This research examined whether multidimensional mindful self-care predicts healthcare practitioner clinical empathy.

**Methods:**

A cross-sectional predictive design was employed with a sample of 153 healthcare practitioners in Saudi Arabia. Participants filled out the Arabic versions of the Multidimensional Clinical Empathy Scale (M-CES) and the Mindful Self-Care Scale–Brief (MSCS-Brief). We used Pearson correlations, multiple regression, and structural equation modelling (SEM) to look at the data. To ensure model parsimony, professional experience was retained as the sole control variable in the structural model.

**Results:**

The relational and structural self-care dimensions had the most robust positive correlations with total clinical empathy. Multiple regression analysis revealed that self-care dimensions collectively accounted for 28.5% of the variance in empathy (R² = 0.285, *p* < .001). Supportive structure and supportive relationships were identified as significant positive predictors, whereas physical care exhibited a negative association. The structural equation model demonstrated acceptable fit χ²(43) = 92.82, CFI = 0.91, RMSEA = 0.09) and confirmed a significant direct association of mindful self-care on clinical empathy (β = 0.51, *p* < .001). When self-care was taken into account, professional experience did not substantially predict empathy (β = 0.14, *p* = .076).

**Conclusion:**

The results show that multidimensional self-regulatory self-care activities have a stronger association with clinical empathy than professional experience. Relational and structural self-care strategies seem to be especially important for sustaining empathic involvement in therapy. These findings support a process-oriented perspective of empathy as a cultivable professional skill rooted in deliberate self-regulation.

## Background

Worldwide, healthcare practitioners face substantial professional demands characterised by workloads, emotional challenges, and patient care responsibilities. Recent evidence indicates that stress prevalence among healthcare workers reaches 25%–40%, substantially exceeding that of comparable professions [1]. In this study, mindful self*-*care is conceptualised as a multifaceted self-regulatory framework that includes behavioural, relational, cognitive, and reflective activities designed to maintain psychological and professional well-being [2, 3]. Clinical empathy is defined as a multidimensional professional capacity involving affective attunement, cognitive perspective-taking, and behavioural expressions of understanding within therapeutic interactions [4, 5]. Self-care and clinical empathy are essential components in the professional performance, job engagement, and burnout susceptibility of healthcare practitioners. Emotional labour has been shown to significantly influence employee outcomes in healthcare contexts [12]. Self-care is widely conceptualised as a multidimensional psychological regulation process [20].

Workplace stress and emotional expectations have frequently been linked to burnout and decreased professional well-being among healthcare practitioners [2–8]. Although self-care has been identified as a protective factor against stress-related outcomes, much of the existing literature has concentrated on burnout and psychological distress rather than investigating whether multidimensional mindful self-care is associated with clinical empathy in therapeutic settings. Healthcare professionals in therapeutic settings increasingly face persistent emotional demands, increasing their risk of psychological distress, emotional tiredness and professional burnout. Global evidence reveals that the prevalence of burnout among healthcare professionals varies from 30% to over 60%, with emotional tiredness being the predominant characteristic [6, 7]. A thorough systematic assessment of physicians in 45 countries found burnout-prevalence estimates between 0% and 80.5%, highlighting the problem’s size and variation across healthcare systems [10]. Therapists in medicine, nursing, psychotherapy, and allied health sectors are required to demonstrate clinical empathy through affective attunement, cognitive perspective-taking, and regulated emotional responsiveness in therapeutic interactions, attunement, and affect management. This persistent emotional participation is called emotional labour, which involves regulating emotions to satisfy professional and organisational standards [8]. Empirical studies consistently demonstrate that high emotional labour is associated with increased emotional exhaustion, depersonalisation, reduced job satisfaction and compromised quality of care [9, 13]. The purpose of this study was to examine how self-care and clinical empathy are associated in therapeutic contexts affect healthcare professionals’ mental health and quality of life. We also sought to help clarify predictive correlations to improve evidence-based therapies and training programmes that emphasise self-care and clinical empathy for sustained high-quality therapeutic practice.

Empirical research has repeatedly demonstrated that mindful self-care and self-compassion are negatively correlated with burnout and compassion fatigue among healthcare populations [14, 15, 16]. Moreover, self-compassion has been associated with enduring compassion for others when psychological well-being is maintained [19]. Nonetheless, few studies have investigated whether multidimensional self-care forecasts clinical empathy as a professional competency rather than only alleviating misery.

In this conceptual framework, mindful self-care is recognised as a multifaceted construct that includes physical, psychological, relational, and structural dimensions [23]. Imbalance in these areas has been linked to healthcare providers’ decreased ability to adjust.

While thoughtful self-care has been well investigated as a protective factor against burnout and psychological distress among healthcare workers, there has been relatively less focus on its possible function in forecasting adaptive professional qualities, including clinical empathy. Much of the existing literature conceptualises self-care within deficit-oriented frameworks that prioritise symptom reduction over functional professional development.

Clinical empathy signifies not only the lack of tiredness or suffering, but a controlled, enduring professional competence that amalgamates emotional attunement, cognitive perspective-taking, and behavioural responsiveness within therapeutic exchanges. Empirical evidence examining whether multidimensional self-care domains contribute differentially to the development of clinical empathy within a structured predictive model remains limited. Addressing this gap is essential for advancing towards a process-oriented understanding of how deliberate self-regulatory practices may shape sustainable empathic functioning in therapeutic contexts.

### Aim & hypotheses

The research sought to investigate the correlation between multidimensional mindful self-care activities and clinical empathy among healthcare practitioners. The study aimed to ascertain the positive correlation between the psychological and relational dimensions of self-care and clinical empathy, to evaluate the distinct associations of physical self-care, and to determine the persistence of these associations as statistically significant after controlling for professional experience through regression and structural equation modelling.

#### Hypotheses

- H1: Multidimensional self-care domains are significantly associated with clinical empathy.
- H2: Psychological and relational self-care domains demonstrate stronger positive associations with clinical empathy compared to physical self-care.
- H3: Professional experience shows a limited or non-significant association with clinical empathy when self-care is included in the structural model.

## Methods

### Study design

This study employed a cross-sectional predictive–correlational design to examine the associations between multidimensional mindful self-care practices and clinical empathy among healthcare practitioners in the Kingdom of Saudi Arabia. Data were collected using standardised psychometric instruments and analysed through multivariate regression and structural equation modelling (SEM). The study was conducted in accordance with the principles of the Declaration of Helsinki.

**Fig. 1:**
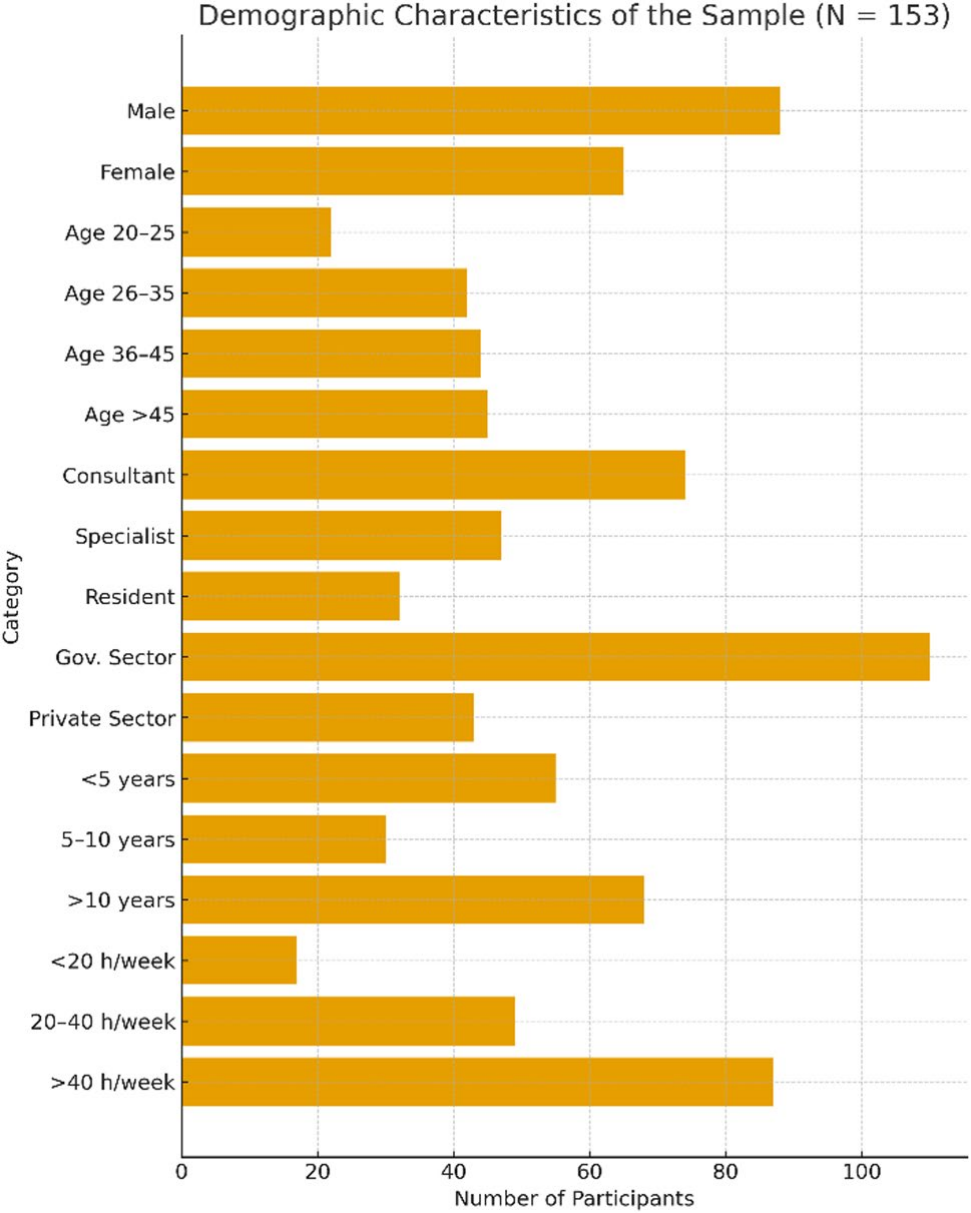
Sample Characteristics (*N* = 153)

Table 1; Fig. 1 summarise the demographic characteristics of the participants (*N* = 153). Nearly half of the sample was categorised as consultants, and the majority consisted of male healthcare providers employed by government entities. The majority of the participants had been in the field for over a decade and worked for over 40 h a week. The results of structural equation modelling, which are presented in the following sections, can be better understood considering these distributions.

**Table 1:** Demographic characteristics of the study sample (*N* = 153)

Variable	Category	*N*	%
Gender	Male	88	57.5
	Female	65	42.5
Age	20–25 years	22	14.4
	26–35 years	42	27.5
	36–45 years	44	28.8
	Older than 45 years	45	29.4
Job Classification	Consultant	74	48.4
	Specialist	47	30.7
	Resident	32	20.9
Workplace	Governmental Healthcare Sector	110	71.9
	Private Healthcare Sector	43	28.1
Experience	< 5 years	55	35.9
	5–10 years	30	19.6
	> 10 years	68	44.4
Weekly Working Hours	< 20 h/week	17	11.1
	20–40 h/week	49	32.0
	> 40 h/week	87	56.9

### Setting and participants

The study was conducted in the Kingdom of Saudi Arabia across both public and private healthcare sectors, including hospitals, primary care centres, outpatient clinics, and private healthcare facilities. Healthcare practitioners were recruited using a structured online survey developed through Google Forms. The survey link was distributed electronically through professional networks, physicians, healthcare staff, and administrative contacts within hospitals and clinical institutions to facilitate access to eligible participants. Data collection took place from September through November of 2025. Inclusion criteria were: (1) current employment in a healthcare or therapeutic role within public or private healthcare institutions in Saudi Arabia, (2) direct involvement in patient care, and (3) provision of informed consent prior to participation. Exclusion criteria included non-clinical administrative roles, incomplete survey responses, and failure to meet eligibility criteria.

Of the invited participants (approximately 200), 153 completed the survey, representing an estimated response rate of about 75%. After screening for completeness and eligibility, valid responses were retained for statistical analysis. Participation was voluntary and anonymous.

The sample consisted predominantly of experienced healthcare practitioners working in structured institutional environments in Saudi Arabia; nearly half of the participants held consultant-level positions, and a substantial proportion reported more than ten years of professional experience. Most were employed in governmental healthcare institutions and worked more than 40 h per week. While this composition enhances the contextual relevance of the findings within institutional healthcare settings, it may limit generalisability to early-career professionals, private practice contexts, or healthcare systems in other countries. These characteristics should therefore be considered when interpreting the external validity of the present study.

### Data Collection Procedure

Data were collected using a structured, self-administered online questionnaire developed through Google Forms. The survey link was distributed electronically to eligible healthcare practitioners through professional networks, institutional contacts, and healthcare administrators across public and private healthcare institutions in Saudi Arabia. The survey consisted of an introductory page explaining the purpose of the study, confidentiality assurances, and participants’ rights. Electronic informed consent was obtained before particpants accessed the questionnaire. Participation was voluntary and anonymous. No identifying information was collected. To enhance response rates, reminder messages were circulated periodically during the data collection period. Completed responses were screened for completeness and eligibility prior to statistical analysis. Incomplete submissions and responses failing eligibility criteria were excluded from the final dataset.

### Measurement

The participants completed a brief demographic questionnaire, from which we could characterise the sample and assess differences in mindful self-care and clinical empathy across professional groups. Gender, years of experience, job setting, degree of education and type of professional development were the main variables. We used two standardised measures: the Mindful Self-Care Scale—Brief, which evaluates six aspects of mindful self-care, and.

#### Mindful Self-Care Scale – Brief (MSCS-Brief)

The initial instrument was the Mindful Self-Care Scale – Brief (MSCS-Brief) [15]. Mindful self-care practices were assessed using the Mindful Self-Care Scale – Brief (MSCS-Brief). The instrument consists of 24 items measuring multiple dimensions of self-care practices, including physical, supportive relationships, mindful awareness, self-compassion, and mindful relaxation. Participants rate items using a Likert-type scale reflecting frequency of engagement in self-care behaviours, with higher scores indicating greater engagement in mindful self-care practices.

#### Multidimensional Clinical Empathy Scale (M-CES)

The Multidimensional Clinical Empathy Scale (M-CES) [16] is a 26-item instrument rated on a 5-point Likert scale and designed to assess professional clinical empathy across four core dimensions: empathetic connections, valuing empathy, genuine concern/empathetic behaviours and perspective taking. Total scores are computed by summing all items and deriving an overall mean, with subscale means calculated separately to generate a multidimensional empathy profile capturing cognitive, affective and behavioural components of clinical empathy.

#### Validity and reliability

Internal consistency was strong for both instruments in the present sample. The MSCS-Brief showed excellent internal consistency (Cronbach’s α = 0.91), with subscale coefficients between 0.78 and 0.88 and item-total correlations between 0.42 and 0.67. The M-CES demonstrated similarly strong internal consistency (Cronbach’s α = 0.93), with subscale alphas between 0.80 and 0.89. Confirmatory factor analysis supported the proposed factor structures for both measures, with factor loadings ranging from 0.58 to 0.82 and acceptable model fit indices (CFI = 0.95; RMSEA = 0.06).

Given that structural equation modelling was employed in the present study, establishing measurement adequacy within the current sample was methodologically essential. The satisfactory internal consistency coefficients and confirmatory factor analysis results provide empirical support for the latent structure of both instruments. The observed factor loadings and acceptable fit indices indicate that the measurement models were psychometrically appropriate for subsequent structural modelling.

### Translation of study measures

A concise translation and cultural adaptation process was executed for MSCS-Brief and M-CES. Two bilingual specialists conducted distinct forward translations that were amalgamated into a cohesive Arabic draft. This version was then back-translated by independent translators, and differences were rectified using linguistic and expert evaluations to ensure conceptual comparability. Initial reliability assessments (e.g. kappa and ICC indices) demonstrated satisfactory consistency, validating the appropriateness of the Arabic versions for application in the current study.

### Statistical analysis

Statistical analyses were performed using SPSS and AMOS/JASP. Descriptive statistics (means, standard deviations and frequencies) were used to summarise the sample characteristics and main study variables. Normality assumptions were examined using skewness and kurtosis indices. Pearson’s correlation coefficient was computed to assess the association between mindful self-care and clinical empathy. Multiple linear regression analyses were conducted to identify key predictors of overall clinical empathy scores. Finally, structural equation modelling (SEM) was used to evaluate the proposed relationships among professional experience, mindful self-care and empathy, relying on standard model fit indices (CFI, TLI, RMSEA and R²). Statistical significance was set at *p* < .05. To ensure model parsimony and avoid saturation, only professional experience was retained as a control variable in the final structural model.

## Results

### Level of self-care practices among healthcare practitioners in therapeutic contexts

Table 2 displays the descriptive data for the six subscales of mindful self-care and total scores of the MSCS-Brief. Mean scores across domains varied from 11.34 for Mindful Awareness (SD = 2.47) and 12.14 for Mindful Relaxation (SD = 3.54) to 15.32 for Supportive Relationships (SD = 3.09) and 14.93 for Supportive Structure (SD = 2.73), demonstrating a comparatively greater engagement in relational and structural self-care practices than in mindfulness-based relaxation and awareness. The Self-Compassion and Purpose subscale exhibited a notably high mean (M = 14.55, SD = 2.65), indicating that numerous practitioners experience a significant feeling of self-kindness and purpose in their everyday activities.

**Table 2:** Descriptive statistics for mindful self-care subscales (MSCS-Brief)

Scale	Mean	Std. Deviation	Skewness	Std. Error of Skewness
Mindful Relaxation	12.144	3.536	0.059	0.196
Physical Care	12.869	3.228	0.395	0.196
Self-Compassion and Purpose	14.549	2.651	−0.714	0.196
Supportive Relationships	15.320	3.088	−0.626	0.196
Supportive Structure	14.928	2.729	−0.254	0.196
Mindful Awareness	11.340	2.471	−0.347	0.196
MSCS-Brief (total score)	81.150	11.574	0.096	0.196

The average MSCS-Brief score was 81.15 (SD = 11.57), indicating a predominantly moderate to moderately high level of self-care practice among the surveyed healthcare professionals. The skewness coefficients for the majority of subscales were minimal (|skew| < 0.75), with Self-Compassion and Purpose (skew = − 0.71) and Supportive Relationships (skew = − 0.63) exhibiting moderate negative skewness, indicating a slight inclination towards elevated scores in these areas. Distribution indices endorse the application of parametric analyses to future inferential tests.

### Level of clinical empathy among healthcare practitioners during therapeutic care

In Table 3,across all subscales and the M-CES total score, the means are comparatively elevated and exhibit moderate to strong negative skewness (skewness = -1.05 to -1.52). This pattern suggests that healthcare practitioners tend to cluster toward the upper end of the empathy scale, with relatively few low-empathy responses. The standard deviations are small compared to their respective means, suggesting minimal variability and a predominantly uniform level of strong clinical empathy within the sample.

**Table 3:** Descriptive statistics for clinical empathy subscales (M-CES)

Scale	Mean	SD	Skewness	SE Skewness
Empathetic Connections	17.275	2.838	-1.070	0.196
Valuing Empathy	22.804	2.829	-1.095	0.196
Empathetic Behaviours	41.092	4.758	-1.519	0.196
Perspective Taking	31.209	3.786	-1.165	0.196
M-CES (total score)	112.379	12.018	-1.049	0.196

### Associations between self-care dimensions and clinical empathy

Correlation analysis showed that relational and structural self-care dimensions were most strongly associated with overall empathy, whereas physical care demonstrated weak or negative associations. As shown in Table 4.

**Table 4:** Pearson correlations between self-care dimensions and empathy dimensions

Self-Care Dimensions	Empathetic Connections	Valuing Empathy	Empathetic Behaviours	Perspective Taking	M-CES
Mindful Relaxation	0.231**	0.167*	0.146	0.163*	0.203*
Physical Care	0.023	− 0.090	− 0.224**	− 0.159	− 0.155
Self-Compassion & Purpose	0.254**	0.263**	0.260**	0.272***	0.311***
Supportive Relationships	0.358***	0.214**	0.370***	0.330***	0.385***
Supportive Structure	0.396***	0.220**	0.336***	0.374***	0.396***
Mindful Awareness	0.224**	0.184*	0.297***	0.295***	0.307***

To determine which specific self-care dimensions independently predict overall clinical empathy at the observed-variable level, a multiple regression analysis was conducted as a preliminary predictive step before testing the full structural model.

Multiple regression analysis indicated that self-care dimensions were associated with 28.5% of the variance in overall empathy scores in overall empathy (R² = 0.285, *p* < .001). Supportive structure and supportive relationships emerged as significant positive predictors, whereas physical care showed a significant negative association. Other dimensions were not statistically significant. As presented in Table 5.

**Table 5:** Multiple regression predicting overall clinical empathy from self-care dimensions

Predictor	B	SE	β	t	*p*	95% CI (Lower–Upper)	Tolerance	VIF
(Intercept)	84.630	6.098	—	13.878	< 0.001	72.579–96.681	—	—
Mindful Relaxation	0.446	0.265	0.131	1.680	0.095	-0.079–0.971	0.797	1.255
Physical Care	–1.066	0.274	–0.286	–3.890	< 0.001	-1.608 to -0.524	0.898	1.114
Self-Compassion and Purpose	0.468	0.371	0.103	1.262	0.209	-0.265–1.201	0.727	1.375
Supportive Relationships	0.868	0.334	0.223	2.598	0.010	0.208–1.529	0.659	1.517
Supportive structure	1.068	0.393	0.243	2.714	0.007	0.290–1.845	0.609	1.641

### Does self-care significantly predict empathy among healthcare practitioners when controlling for professional experience?

To examine the hypothesised relationships at the latent construct level and to evaluate overall model fit beyond observed-variable regression, structural equation modelling (SEM) was performed.

Confirmatory factor analysis (CFA) was conducted to evaluate the adequacy of the measurement model prior to testing the structural relationships.

As shown in Table 6, all standardised factor loadings were statistically significant and ranged from moderate to high, supporting the convergent validity of the constructs. The indicators loaded appropriately on their respective latent constructs, supporting the convergent validity of the measurement model.

**Table 6:** Standardised factor loadings for the measurement model (CFA)

Construct	Indicator	β	*p*
Self-Care	Physical Care	0.20	0.037
	Self-Compassion & Purpose	0.65	< 0.001
	Supportive Relationships	0.69	< 0.001
	Supportive Structure	0.72	< 0.001
	Mindful Awareness	0.65	< 0.001
Empathy	Valuing Empathy	0.72	< 0.001
	Empathetic Behaviours	0.85	< 0.001
	Perspective Taking	0.87	< 0.001

The proposed structural model demonstrated an acceptable fit to the data. The chi-square test was statistically significant, χ²(43) = 92.82, *p* < .001. Incremental fit indices indicated adequate fit, with CFI = 0.91 and TLI = 0.88. The RMSEA value was 0.09 (95% CI [0.06, 0.11]), and SRMR was 0.08. Although the chi-square was significant, which is common in moderately sized samples, the overall pattern of fit indices suggests that the model provides a reasonable representation of the observed covariance structure.

Self-care was significantly associated with empathy (β = 0.51, B = 0.59, SE = 0.16, z = 3.78, *p* < .001), indicating that higher levels of self-care were associated with greater emotional engagement among healthcare practitioners. Professional experience showed a positive but statistically non-significant association with empathy (β = 0.14, B = 0.29, SE = 0.16, z = 1.77, *p* = .076), suggesting that experience did not uniquely predict empathy when self-care was included in the model.

Table 7 presents the standardised structural path coefficients of the final structural equation model. Mindful self-care demonstrated a significant positive association with clinical empathy (β = 0.51, *p* < .001), indicating that higher levels of self-regulatory self-care practices were associated with stronger empathic engagement. In contrast, professional experience did not show a statistically significant association with clinical empathy (β = 0.14, *p* = .076), suggesting that professional experience did not demonstrate a statistically significant independent association with empathy when self-care was included in the model.

**Table 7:** Standardised Structural Path Estimates for the Final SEM Model

Path	β	*p*
Self-Care → Clinical Empathy	0.51	< 0.001
Professional Experience → Clinical Empathy	0.14	0.076

Discriminant validity was supported, as the HTMT ratio between self-care and empathy was 0.53, below the recommended threshold of 0.85.

**Fig. 2:**
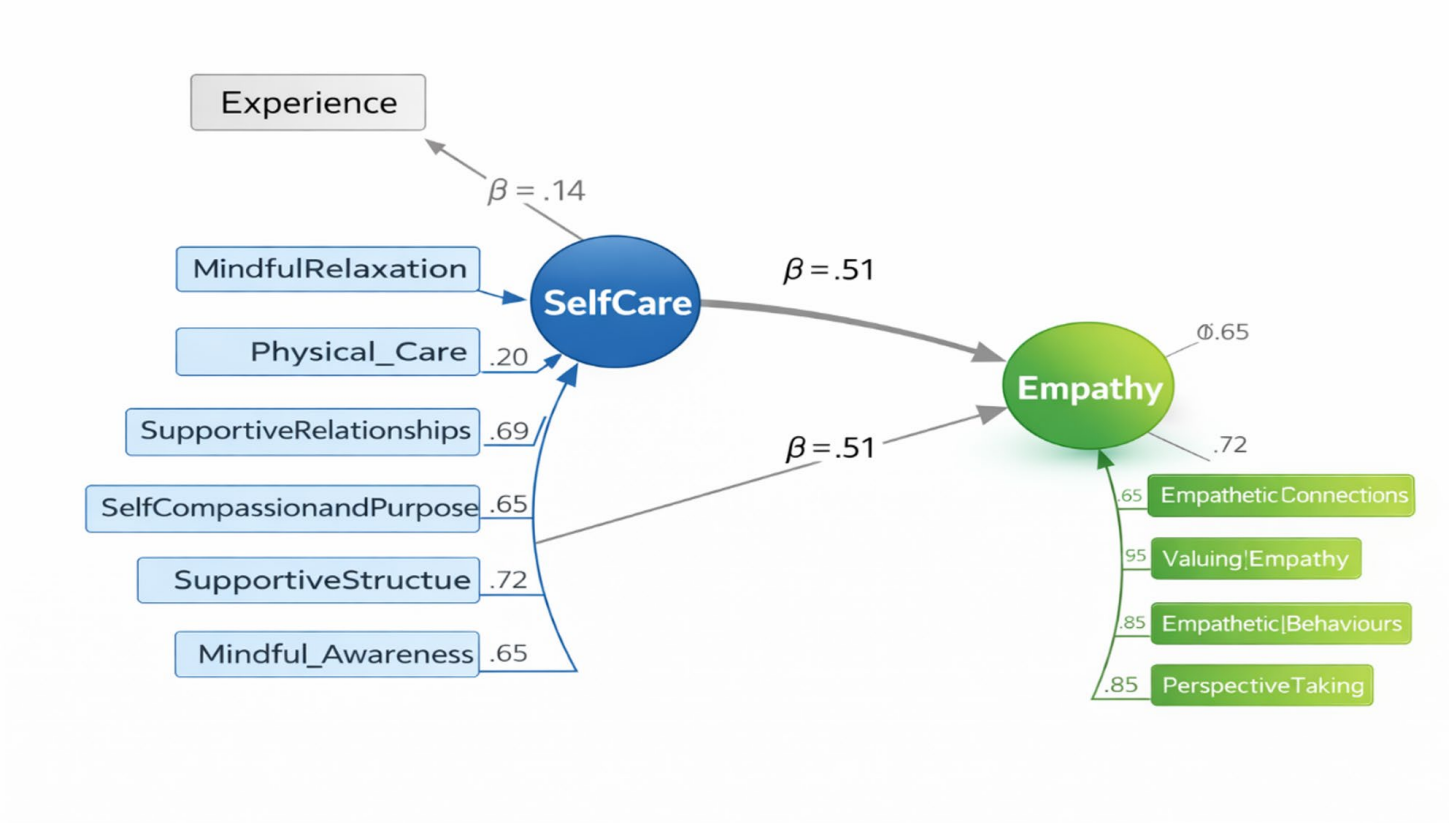
Structural model of self-care predicting empathy

Figure 2 presents the final structural model examining the predictive relationship between mindful self-care and clinical empathy, controlling for professional experience. The standardised estimates indicate a substantial positive association of self-care on empathy (β = 0.51, *p* < .001), whereas professional experience showed a non-significant association (β = 0.14, *p* = .076). The model demonstrates adequate overall fit and is consistent with the proposed hypothesis that self-care is positively associated with clinical empathy.

## Discussion

With especially high ratings in self-compassion and purpose, supportive connections and structure, healthcare practitioners demonstrated consistently high levels of attentive self-care across all six categories [2, 6, 7, 13, 17]. These descriptive findings indicate relatively strong engagement in relational and structural self-care domains within the present sample. However, the cross-sectional design does not allow conclusions regarding the underlying causes of these patterns [4, 18]. Previous research has linked self-compassion with reduced burnout and improved wellbeing among healthcare professionals [11]. Garcia-Campayo et al. [14] discovered that professionals with superior psychological well-being exhibit sustained and improved empathy. In the present study, clinical empathy scores were consistently high across all measured dimensions. Given the cross-sectional nature of the study, these findings reflect the current distribution of empathy levels within the sample and should not be interpreted as evidence regarding changes in empathy over time.

Most self-care domains positively correlated with clinical empathy, with supporting structures, supportive connections and mindful awareness being the strongest. These findings support previous research showing that empathy is relational and context-dependent [18, 27]. Self-compassion and mindfulness were linked to improved perspective-taking and empathy, supporting Garcia-Campayo et al.’s findings [19]. Most self-care domains were positively correlated with clinical empathy, with supportive structure, supportive relationships and mindful awareness demonstrating the strongest associations. Physical care showed weak or negative correlations. The negative association observed for physical self-care should be interpreted cautiously. One possible explanation is that physical self-care behaviours, when examined independently from relational and structural domains, may not directly translate into empathic engagement within clinical interactions. It is also plausible that shared variance among self-care dimensions may have resulted in a suppression effect within multivariate analyses. Additionally, unmeasured contextual factors such as occupational stress, burnout severity, or role overload may partially account for this association. Given the cross-sectional design, these interpretations remain tentative and warrant further longitudinal investigation. These patterns are consistent with prior literature linking relational self-regulatory resources to empathic engagement [21]. The findings support Butler et al.’s findings [2] using the multidimensional self-care approach, which prioritises balance over frequency.

Supportive structure and connections were the strongest positive predictors of clinical empathy in regression and structural equation models, but physical care was a negative predictor when psychosocial factors were controlled [13, 22]. indicating a statistically significant association between self-care and empathic engagement within this sample.

Regarding whether empathy may be conceptualised as a professional capacity associated with self-regulatory processes. The present findings indicate that multidimensional self-care was statistically associated with clinical empathy within this sample. The strength of associations observed for supportive structure and supportive relationships suggests that relational and organisational self-regulatory practices were more strongly linked to empathy than physical self-care behaviours. These results should be interpreted strictly as associative patterns within a cross-sectional framework and do not imply directional or causal relationships [4, 19].

In the final structural model, professional experience did not significantly predict clinical empathy when self-care was included. Within the present sample, years of practice were not statistically associated with empathy when self-care was included. Rather, self-care dimensions were more strongly associated with empathy within the present model [4, 19, 14, 23].

Weekly working hours were not significantly associated with clinical empathy in the present analyses. Although workload is often discussed in relation to emotional exhaustion, the current data do not allow conclusions regarding indirect or mediating mechanisms. Future research may examine whether occupational stress variables influence empathy through self-regulatory processes [24–27].

In correlational and predictive studies, psychological and relational self-care behaviours were connected to clinical empathy more than behavioural or somatic practices. Demographic variables accounted for comparatively little variance in empathy, suggesting that contextual and self-regulatory processes were more strongly associated within the present model [28, 29].

Overall, the findings indicate that multidimensional self-care, particularly relational and structural domains, was significantly associated with clinical empathy at both observed and latent levels of analysis. Professional experience did not demonstrate a statistically significant independent association when self-care was included in the structural model. These results suggest that self-regulatory practices may be more strongly related to empathy than years of practice within the present sample. However, due to the cross-sectional design, interpretations should remain limited to associative relationships rather than causal conclusions.

## Conclusion

These findings indicate that mindful self-care was significantly associated with clinical empathy among healthcare practitioners. Supportive structure and supportive relationships demonstrated the strongest positive associations within the present structural model. However, physical care was found to have a negative correlation, highlighting the importance of maintaining a healthy balance between psychological and relational self-regulation techniques and physical routines. The findings suggest the potential importance of including organised self-care and relational support in professional development programmes to improve clinical empathy in therapeutic settings, reinforced by the fact that demographic characteristics have a minor influence on the development of empathy. This finding highlights the importance of targeted training and continuous professional growth.

## Limitations and strengths

This study has several methodological limitations that should be acknowledged. First, the cross-sectional design precludes causal inferences. Although structural equation modelling allows examination of predictive associations, the temporal direction of association between mindful self-care and clinical empathy cannot be definitively established. Longitudinal or experimental designs would be required to confirm causal pathways. Second, the reliance on self-report measures introduces the possibility of response bias, including social desirability and common method variance. Because both predictor and outcome variables were assessed using the same measurement method at a single time point, shared method association may have inflated observed associations. Third, although the sample included practitioners from both public and private healthcare sectors, the study was conducted within a single national context, which may limit generalisability to other healthcare systems. Future research should incorporate longitudinal designs, multi-source assessments (e.g., patient-reported empathy), and objective workload indicators to strengthen causal interpretation and reduce method bias.

Additionally, unmeasured variables such as burnout severity, occupational stress, and resilience may have influenced the observed associations. All interpretations are limited to statistically observed associations within the present sample and should not be generalised beyond similar institutional healthcare contexts without further replication.

## Recommendations

Based on the present findings, several focused recommendations can be derived. The results demonstrated that supportive structure and supportive relationships were the strongest positive predictors of clinical empathy in both regression and structural modelling analyses. Therefore, professional development programmes should prioritise structured self-regulation practices and relational support strategies. Training initiatives may consider emphasising relational and structural self-care practices identified in the present findings, as these dimensions were empirically associated with stronger empathic engagement. At the organisational level, healthcare institutions should foster structured work environments and peer-support systems that sustain relational and psychological resources linked to empathy. Future research should adopt longitudinal designs and examine mediating mechanisms such as burnout and resilience to further clarify the dynamics underlying the self-care–empathy relationship.

## Data Availability

All data supporting the findings of this study are available from the corresponding author upon request. Because of ethical restrictions involving human participants’ privacy, data cannot be made publicly available, but can be shared with qualified researchers upon request.

## Abbreviations

MSCS-Brief: Mindful Self-Care Scale – Brief
M-CES: Multidimensional Clinical Empathy Scale
SEM: Structural Equation Modelling
CFI: Comparative Fit Index
RMSEA: Root Mean Square Error of Approximation
β: Standardised Beta Coefficient
